# The changing face of head and neck cancer: are patients with human papillomavirus-positive disease at greater nutritional risk? A systematic review

**DOI:** 10.1007/s00520-022-07056-9

**Published:** 2022-04-27

**Authors:** Anna Edwards, Teresa Brown, Brett G. M. Hughes, Judy Bauer

**Affiliations:** 1 School of Human Movements and Nutrition Sciences, University of Queensland, Brisbane, QLD Australia; 2 Nutrition & Dietetics, Toowoomba Hospital, Darling Downs Health, Toowoomba, QLD Australia; 3 Nutrition & Dietetics, Royal Brisbane and Women’s Hospital, Metro North Hospital and Health Service, Brisbane, QLD Australia; 4 Cancer Care Services, Royal Brisbane and Women’s Hospital, Metro North Hospital and Health Service, Brisbane, QLD Australia; 5 School of Medicine, University of Queensland, Brisbane, QLD Australia; 6 Nutrition, Dietetics & Food, School of Clinical Sciences, Monash Health, Monash University, Victoria, Australia

**Keywords:** Human papillomavirus, Oropharyngeal cancer, Malnutrition, Weight loss, Nutrition, Nutrition support

## Abstract

**Purpose:**

Human papillomavirus (HPV) is now the primary cause of oropharyngeal head and neck cancer (OPC) worldwide; yet limited research has examined the effect of HPV-positive status (OPC+) on nutrition outcomes. This study aims to determine the impact of HPV status on nutritional outcomes for adult patients with OPC undergoing any treatment modality.

**Methods:**

A systematic literature review was conducted up to and including July 2021 of PubMed, Embase, CENTRAL, CINAHL, and Web of Science to identify studies conducted in adults (>18 years) with known OPC reporting on any outcome(s) related to nutrition, according to HPV status (OPC+ versus OPC−). Bias was assessed using QUIPS tool, with certainty of evidence assessed using GRADE system.

**Results:**

Six studies (total *n* = 635) all at moderate-high risk of bias were included. Three studies reported on weight change (*n* = 255), three feeding tube dependency (*n* = 380), three feeding tube timing of placement (prophylactic or reactive) and/or utilisation (*n* = 255), two nutritional (energy and/or protein) intake (*n* = 230), and one nutritional status (*n* = 83). Patients with OPC+ may experience greater weight loss, may have higher utilisation of reactive feeding tubes (both GRADE low certainty, downgraded due to serious bias and imprecision), and may have lower feeding tube dependency rates (GRADE low certainty, downgraded due to serious bias and inconsistency) versus OPC− . It is uncertain whether nutritional intake and nutritional status differed between populations (GRADE very low certainty, downgraded due to serious bias and very serious imprecision).

**Conclusion:**

Further, high-quality research is needed to understand optimal nutritional care practices for patients with OPC + to achieve positive health outcomes into survivorship.

**Supplementary Information:**

The online version contains supplementary material available at 10.1007/s00520-022-07056-9.

## Introduction

The negative impact of malnutrition on patients with oropharyngeal squamous cell carcinoma (OPC) [1–3] is now well established with the causes being multifactorial [4, 5]. Increased rates of treatment interruptions [4, 6], treatment toxicities [1], need for enteral (tube) feeding [7, 8], long-term tube feeding dependency [9], reduced quality-of-life (QoL) [10], treatment tolerance [4], and ultimately survival [5] are some of the known sequelae of malnutrition development, which  may worsen with the intensive multimodal treatment regimens currently recommended to induce remission. Significant weight loss (≥ 5% in 1 month or ≥ 10% at 3-months post-treatment completion) has been seen in up to 80% and malnutrition in up to 88% of patients during treatment [11–13]. Oncogenic human papillomavirus (HPV) is now recognised as the primary cause of a rapid increase in patients diagnosed with OPC worldwide, which is now considered to be at epidemic levels [14, 15].

HPV-positive OPC (OPC+) represents a distinct tumour entity compared to HPV-negative OPC (OPC−), displaying unique histopathological, biological, and clinical characteristics [15]. The American Joint Committee on Cancer (AJCC) eighth-edition Tumour-Node-Metastasis (TNM) classification system now distinguishes between these two histopathological sub-types, and recommends separate staging models [16]. Patients with OPC+ are often younger, non-smokers, more likely to be overweight and/or obese, and are less likely to present with pre-treatment weight loss due to the absence of tumour-related dysphagia or odynophagia limiting oral intake [17–19]. Patients with OPC+ have a better prognosis with improved response to treatment and favourable survival outcomes compared to those with OPC− , despite a trend for higher grade and more advanced nodal disease [20–22]. Despite the significantly improved prognosis and greater sensitivity to current high-dose chemoradiation treatment regimens frequently used to treat OPC+ , higher rates of treatment related toxicities and chronic functional and psychological status impacts affecting QoL into the survivorship phase have been reported [3, 15, 18, 23]. Recent attempts to de-escalate the high-dose radiation with cisplatin in an attempt to reduce treatment burden for this population have not yet proved successful [24]. Therefore, the risk of nutritional decline and long-term treatment-related morbidity for patients with OPC+ remains high.

The rising prevalence of OPC+ is expected to continue [15]. Limited nutritional research has been conducted specific to this population, despite evidence to suggest higher rates of weight loss and the associated negative impacts on morbidity and mortality [3, 18]. Adapting and optimising current nutrition intervention protocols and strategies for head and neck cancer (HNC) to this unique subset of patients is imperative, given the impacts of anti-HPV vaccination programs currently approved to prevent HPV-related cervical, vaginal, and vulvar cancers on OPC+ prevalence will not be known for decades [25]. To our knowledge, this is the first systematic literature review conducted of studies that aims to determine the effect of current treatment regimens (any modality) on nutrition outcomes for patients with OPC+ compared to patients with OPC− .

## Methods

This systematic literature review was undertaken and reported in accordance with the Preferred Reporting Items for Systematic Reviews and Meta-Analysis (PRISMA) guidelines [26] and was registered prospectively on the 13 March 2021 on the PROSPERO International Register of Systematic Reviews (Registration no: CRD42021248974) database.

### Eligibility criteria

The eligibility criteria for studies to be included in this review were formed based on a Population, Intervention, Comparison, and Outcomes (PICO) statement (Supplementary Table 1). All study types with the exception of review articles or conference abstracts were eligible for inclusion if they were published in the English language and (a) included adult patients (> 18 years of age) undergoing any treatment modality for OPC (inclusive of cancers of the base of tongue, soft palate, tonsils and walls of the pharynx), (b) reported HPV status and included patients with OPC+ disease compared to patients with OPC− in analysis, and (c) reported outcome(s) associated with nutritional status and/or nutritional care of these patients. Only full text peer-reviewed journal articles were included. Studies that reported only survival outcomes or treatment-related outcomes (i.e., toxicities, interruptions, QoL) were excluded as these have been reported on elsewhere [27–30]. Studies that contained a mixed HPV-positive HNC population (regardless of whether they included patients with OPC+) or compared patients with OPC+ to a mixed HNC population were also excluded, as research demonstrates HPV presence in other HNC populations does not result in the same improved prognosis and treatment response [31, 32].

### Search strategy

A systematic review of the literature was conducted in the electronic databases PubMed, CINAHL, Embase, CENTRAL, and Web of Science. The search strategy was developed by primary author AE in consultation with a medical librarian. The example search strategy for PubMed can be seen in Supplementary Table 2. Keywords and MeSH search terms related to OPC, HPV, and nutrition were used, with no limitations placed on study type, date of publication, sample size, patient gender, or publication location. Treatment modalities were not specified to allow for all studies reporting nutrition-related outcomes for patients with OPC+ to be considered. The search was conducted by the primary author AE, and included articles published up to and including July 2021. After duplicates were removed, titles and abstracts of the identified relevant articles were screened by AE with reference to the exclusion criteria, with those articles flagged as potentially eligible then screened again by a second author JB. Full text versions of articles screened as eligible were then reviewed by all authors independently, with any disagreement in article eligibility resolved through group discussion to reach an overall consensus to determine final article selection. Reference lists of all included articles were then hand searched, in addition to the searching of the University of Queensland library database, to confirm that all relevant publications of interest were included. 

### Data extraction

Data extraction was performed by the primary author AE followed by an evaluation of data extraction correctness independently undertaken by author JB. Data extracted included study design, year of study design, study population characteristics and number, diagnosis, treatment modality, HPV definition and prevalence, malnutrition prevalence and/or incidence, weight change, feeding tube time of placement, and/or utilisation and/or dependency, along with feeding tube dependency definitions, and nutritional intake. Any confounders present in the articles were also extracted. Respective article authors were contacted if required to obtain missing details.

### Quality assessment and certainty of evidence

Study quality was appraised using the Quality In Prognosis Studies (QUIPS) tool [33]. Individual studies were evaluated with reference to six bias domains: study participation, attrition, prognostic factor and outcome measurement, confounding, statistical analysis, and reporting. Each bias domain consisted of three to nine sub-domains. Each study was assessed against these to determine an overall rating of ‘low’, ‘moderate’, or ‘high’ risk of bias. Inter-rater reliability was assured by the authors AE, TB, and JB independently assigning a quality rating to each study, with any discrepancies in study quality resolved through group consensus. The online Cochrane Review software *Robvis* was used to design risk-of-bias plots [34]. Evidence certainty for the body of evidence was determined using the Grading of Recommendations, Assessment, Development and Evaluation (GRADE) system and corresponding website GRADEPro [35].

### Data analysis and synthesis

Due to the heterogeneity present amongst the identified studies, a meta-analysis could not be performed. Studies have been categorised by their study design, nutrition outcome investigated, definition of nutrition outcome investigated, treatment modalities used, and confounders present. The level of evidence was assessed for each outcome of interest and presented in a narrative summary.

## Results

### Study selection and literature review

**Fig. 1:**
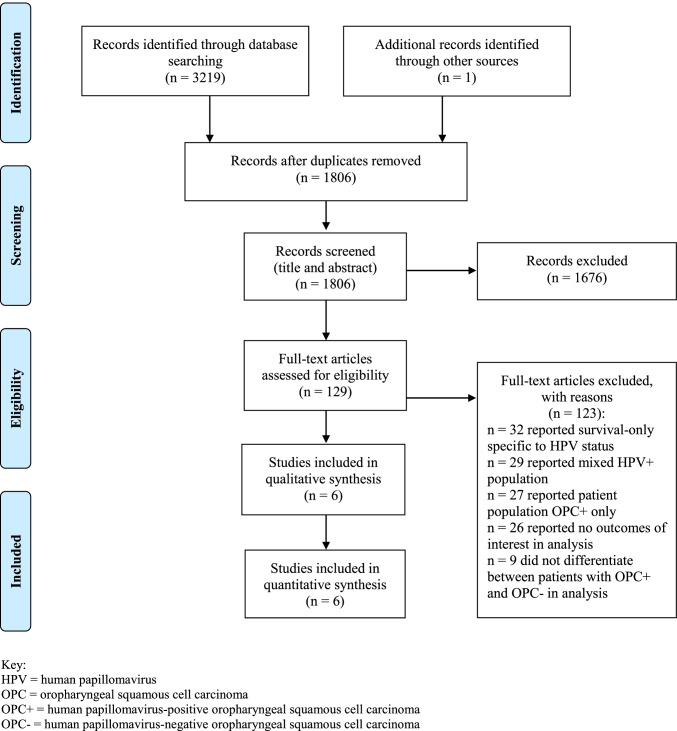
PRIMSA diagram. Key: HPV human papillomavirus, OPC oropharyngeal squamous cell carcinoma, OPC + human papillomavirus-positive oropharyngeal squamous cell carcinoma, OPC − human papillomavirus-negative oropharyngeal squamous cell carcinoma

A total of 4220 studies were identified during the search, with a final six publications [19, 23, 36–40] (total pooled OPC *n* = 635; pooled OPC+ *n = *485) meeting full inclusion criteria (Fig. 1) with results summarised in Table 1. All were observational retrospective cohort studies [19, 23, 36, 38–40]. Three were conducted in the USA [23, 36, 39], one in Canada [38], and two in Australia [19, 40]. Only one study from Australia [19] reported mean BMI of participants at baseline (*n* = 83; OPC+ 29.7 kg/m^2^ (SD ± 6.2) versus OPC− 24.5 kg/m^2^ (SD ± 5.3) (*p* < 0.01)). All studies were conducted in OPC+ populations undergoing surgery and/or chemotherapy and/or radiation [19]. Three studies reported feeding tube (gastrostomy and/or nasogastric) dependency [36, 38, 39], three weight change [19, 23, 40], three feeding tube timing of placement (prophylactic or reactive) and/or utilisation [19, 23, 40], two nutritional (energy and/or protein) intake [19, 39], and one nutritional status (i.e., malnutrition prevalence and/or incidence) [19].

**Table 1:** Characteristics of studies included in this review

Author, Year, Country	Study design, Recruitment timeframe	Number (n)	Population of interest; treatment modalities	Outcome(s) of interest	Outcome definition(s)	Data collection timepoint(s)	Type of analysis; adjustment factors	Results
Bledsoe, 2013, USA	Retrospective observational, 2006–2011	Total *n* = 121 OPC+ *n* = 97 vs OPC− *n* = 24	Patients with stable III–IVb OPC with known HPV status treated with definitive CRT. Patients with prior S or RT were excluded RT: earlier years: 3D conformal and later years IMRT. Delivered as either once-daily fractions of 2 Gy/fraction to a total dose of 70–74 Gy or in twice-daily fractions of 1.2 Gy/fraction to a total dose of 74.4 Gy CT: earlier years fluorouracil with cisplatin and later years high-dose cisplatin	Feeding tube dependency	Rates of gastrostomy tubes in situ at 6-months post-treatment completion	6-months post-RT	Univariate analysis only conducted; nil adjustment factors used in analysis	Feeding tube outcomes: OPC+ less likely to have a gastrostomy in situ at 6-months post-treatment (OPC+ 0%) vs OPC− (24% ( *p* < 0.001))
Harrowfield, 2021, Australia	Retrospective observational, 2013–2016	Total *n* = 83; OPC+ *n* = 70 vs OPC− *n* = 13	Patients with OPC with known HPV status treated with RT CT who had previously consented to the EAT trail. Excluded patients with T1N2 tonsillar carcinomas treated with unilateral treatment RT: method not described; patients were required to undergo at least 60 Gy delivered as regional nodal irradiation CT: agent not described	Weight change, Feeding tube time of placement, Nutritional intake; Nutritional status	Weight change: % loss of weight at each timepoint, categorised as either 5% loss of weight and 10% weight-loss at 3-months post-RT Nutritional Status Change: PG-SGA score change (mean ± SD) and PG-SGA category B/C change (%) Feeding tube time of placement: number of patients with NGT inserted after start RT Nutritional intake: box 2 of PG-SGA	First week of RT, last week of RT, 1-month post-RT, 3-months post-RT	Multivariate analysis; EAT trial intervention, location, time of assessment, date, tumour site, tumour stage, baseline nutritional status	Weight outcomes: OPC+ significantly higher odds of experiencing > 10% loss of weight at 3-months post-RT vs OPC− (OR = 49.68, 95% CI, 2.7–912.86, *p* = < 0.01) Feeding tube outcomes: no significant differences between OPC+ vs OPC − (OR 0.75, 95% CI, 0.22–0.26, *p* = 0.65) at any time point Nutritional intake: no significant differences between OPC+ vs OPC− (*p* = 0.14, 95% CI, − 0.11–0.78) Nutritional status: no significant difference in PG-SGA category (*p* = 0.30 (95% CI, 0.14–1.83) or scores (*p* = 0.44 (95% CI, − 1.22–2.82) for OPC− vs OPC+ at any time point
Marzouki, 2018, Canada	Retrospective observational, 1998–2009	Total *n* = 112; p16 + *n* = 63 vs p16- *n* = 49 (*note: p16 status used as a surrogate for HPV status; i.e., p16*+ = *OPC*+)	Patients with OPC and known HPV status treated with primary surgery ± other treatment modalities S: open surgery and free flap reconstruction RT: method not described; delivered dose not described CT: agent not described	Feeding tube dependency*	Requiring gastrostomy feeding to maintain daily caloric (energy) requirements	End of treatment, 6- and 12-months post-S	Multivariate analysis; p16 status, age, stage, and the percentage of base of tongue and soft palate resection	Feeding tube outcomes: p16+ (OPC+) status did not have a significant effect on feeding tube requirement during treatment (*p* = 0.23) or dependency pre-S (*p* = 0.068); 6-months post-S (*p* = 0.084); or 12-months post-S (*p* = 0.172)
Naik, 2015, USA	Retrospective observational, 1989–2002 (*historical cohort)* 2002–2010 (*intervention cohort)*	Total *n* = 147; OPC+ *n* = 130 vs OPC− *n* = 17	Patients with stable III–IVb OPC with known HPV status treated with definitive CRT S: salvage neck dissection RT: 3D conformal; delivered with once-daily fractions of 2 Gy/fraction to a total dose of 70–74 Gy or in twice-daily fractions of 1.2 Gy/fraction to a total dose of 74.4 Gy CT: fluorouracil with cisplatin or high-dose cisplatin and/or cetuximab	Feeding tube dependency; Nutritional intake	Feeding tube dependency: not defined Nutritional intake: defined as patients requiring nutritional supplements for weight maintenance, or having significant limitations in the types of foods eaten	3-, 6-,12- and 24-months post-RT	Univariate analysis only conducted; nil adjustment factors used in analysis	Feeding tube outcomes: OPC+ had a lower incidence of feeding tube dependency vs OPC− at 3-months (13.2% vs 23.5%, *p* = 0.27); 6-months (6.9% vs 11.8%, *p* = 0.62); 12-months (2.3% vs 11.8%, *p* = 0.10) and 24-months post-RT completion (1.6% vs. 12.5%, *p* = 0.06) Nutritional intake outcomes: OPC+ more likely to have resumed a normal diet at 24-months post-RT and had lower rates of limited diet (8.6% vs. 33.3%, *p* = 0.014) vs OPC−
Vangelov, 2018, Australia	Retrospective observational, 2006–2011	Total *n* = 100; OPC+ *n* = 68 vs OPC− *n* = 10 (HPV status unknown *n* = 22)	Patients with OPC with known HPV status treated with RT ± other treatment modalities S: method not described RT: earlier years: 3D conformal and later years IMRT; delivered dose 5 fractions per week over 6 weeks total Gy received not described) CT: cisplatin (weekly or third-weekly) or high-dose cisplatin or cetuximab	Weight change, Feeding tube time of placement, Feeding tube utilisation	Weight change: defined as weight at week one of RT as baseline and weight in the last week of RT CWL was defined as ≥ 5% loss of weight Feeding tube time of placement: compared prophylactic to reactive tube presence Feeding tube utilisation: the date feeding commenced to date ceased	Baseline (week one of RT) and last week of RT CWL: baseline to 3-months post-RT	HPV status, age, gender, stage, treatment modality, RT dose, neck node irradiation and pre-treatment weight loss	Weight outcomes: OPC+ had significantly higher mean % loss of weight during RT vs OPC− (8.4% vs 6.1%,, *p* = 0.003). CWL was significantly higher in for OPC+ ( *n* = 63/68, 93%) vs OPC− and HPV unknown status ( *n* = 23/32, 72%, *p* = 0.001) OPC+ status and CRT were predictors of CWL on prediction modelling Feeding tube outcomes: OPC+ more often required a feeding tube (*n* = 43/68, 63%) vs OPC− and HPV unknown status ( *n* = 18/32, 56%). Of the 43 patients with OPC+ who received a feeding tube, 27 (63%) had a reactive placement and 16 (24%) prophylactic placement. No patient with OPC+ was using a prophylactically placed gastrostomy at 6 months post-RT completion
Vatca, 2014, USA	Retrospective observational, 2007–2012	Total *n* = 72; OPC+ *n* = 57 vs OPC− *n* = 15	Patients with stable III-IVb OPC with known HPV status treated with definitive CRT Patients with prior S or RT were excluded RT: 3D conformal; delivered dose not described CT: cisplatin or cisplatin and cetuximab or carboplatin and paclitaxel or docetaxel or docetaxel, cisplatin, and 5-fluorouracil	Weight change, Feeding tube utilisation	Weight change: lbs difference at each time point Feeding tube presence and/or utilisation: defined as days of utilisation of a feeding tube	Start of RT, end of RT, 3-months post-RT	HPV status, Race, Smoking status, Nodal staging, RT therapy type (IMRT vs 3D conformal) RT intensity	Weight outcomes: OPC+ had significantly higher rates of weight loss at end (mean 15 lbs vs 8.3 lbs, *p* = 0.015) and 3-months post-RT (mean 23.1 lbs vs 12.6 lbs; *p* = 0.013) vs OPC− Feeding tube outcomes: OPC+ had a longer duration of gastrostomy use during treatment (48.3 days) vs OPC− (mean days 165.4 ± 163.4 vs 117.1 ± 98.2; *p* = 0.39)

*Lbs* pounds, *Gy* grey, *S* surgery, *RT* radiotherapy, *CT* chemotherapy, *CRT* chemoradiotherapy, *TORS* trans-oral robotic surgery, *PG-SGA* patient generated subjective global assessment, *CWL* critical weight loss, *IMRT* intensity-modulated radiation therapy, *OPC* + human papilloma-virus positive oropharyngeal squamous cell carcinoma, *OPC − *human papilloma-virus negative oropharyngeal squamous cell carcinoma, *HPV* human papilloma-virus, *EAT trial* Eating As Treatment (TROG 12.03) trial, *NGT* nasogastric tube

^*^Used as a surrogate for swallowing function, not assessed a primary outcome

### Quality of evidence and certainty of evidence appraisal

**Fig. 2:**
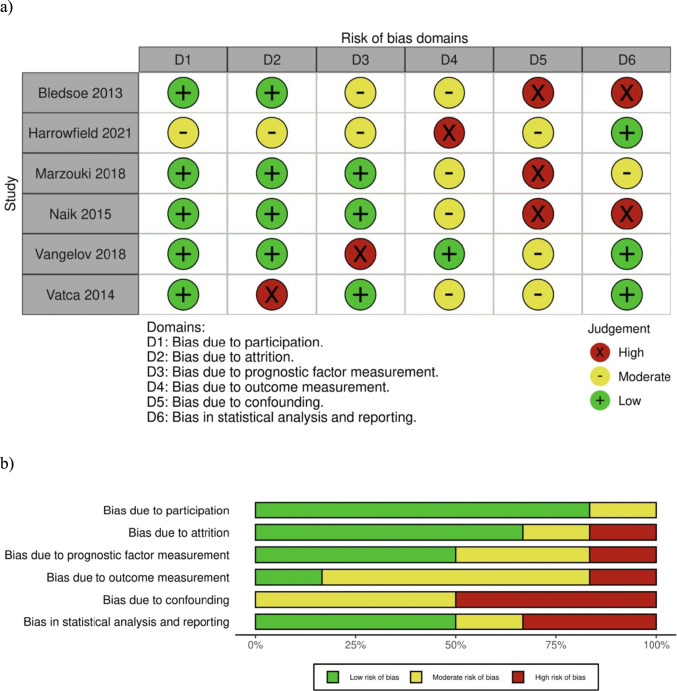
Quality In Prognosis Studies (QUIPS) tool applied utilizing Robovis online software to visualise **a** traffic light plot and **b** weighted summary plot across six risk of bias domains

The QUIPS tool revealed risk of bias was moderate-high for all six studies (Fig. 2a). A ‘high’ risk of bias was due to confounding (three studies), statistical analysis and reporting (two studies) and attrition, prognostic factor measurement, and outcome measurement (one study each respectively). All studies identified demonstrated a moderate-high risk of bias for confounding (Fig. 2b). GRADE certainty of evidence was low for increased rates of weight loss during- and post-treatment, for higher use of reactive feeding tubes (both downgraded due to serious bias and imprecision), and for decreased tube dependency rates (downgraded due to serious bias and inconsistency) when comparing patients with OPC + versus OPC − . It is uncertain whether nutritional intake and nutritional status differed between populations (very low GRADE certainty of evidence, downgraded due to serious bias and very serious imprecision).

### Weight change

Three retrospective cohort studies (pooled *n* = 255)[19, 23, 40] explored the impact of HPV status on weight change either during treatment (all three studies [19, 23, 40]) and/or at 3-months post RT treatment (two studies [19, 23]). Higher rates of weight loss were seen both at end of treatment and at 3-months post-treatment for patients with OPC+ compared to patients with OPC − (GRADE certainty of evidence low, downgraded due to serious bias and imprecision). Harrowfield et al. [19] (total *n* = 83; OPC+ *n* = 70 versus OPC− *n* = 13) demonstrated patients with OPC+ had significantly higher odds of experiencing > 10% loss of weight at 3-months post-RT compared to patients with OPC− (OR = 49.68, 95% CI, 2.7–912.86) *p* < 0.01) despite end of treatment weight loss being less (7.1 SD ± 4.5 versus 8.5 SD ± 5.4 respectively). Patients with OPC+ also demonstrated a clinically important greater decline in the total amount of weight lost overall during treatment (− 5.7% versus − 1.4%, *p* = 0.15) when measured at 3-months post-RT. Vangelov et al. [40] (total *n* = 100; OPC+ *n* = 68, OPC− *n* = 10, HPV unknown *n* = 22) demonstrated patients with OPC+ undergoing RT ± CT experienced significantly higher rates of weight loss at the end of RT compared to patients with OPC− and HPV unknown status (8.4% versus 6.1%, 95% CI, 0.8–3.9, *p* = 0.003). Critical weight loss (defined as  ≥ 5% loss in 1 month) was observed for 93% of patients with OPC+ compared to 60% in patients with OPC− and 77% in patients with HPV unknown status (*p* = 0.011). All but one OPC+ patient lost weight during treatment, with the range of weight loss being 0–17% [40]. When comparing patients based on HPV status using conditional probability modelling (74% accuracy) concurrent chemoradiotherapy and OPC+ status were independent predictors of critical weight loss on univariate analysis (96%, *p* = 0.001 and 98%, *p* = 0.012 respectively) but did not retain significance on multivariate analysis (OR 3.3, 95% CI, 0.9–11.7, *p* = 0.068). Vatca et al. (total *n* = 77, OPC+ *n* = 62 versus OPC− *n* = 15) demonstrated patients with OPC+ undergoing chemoradiotherapy compared to patients with OPC− lost significantly more weight on univariate analysis at end of RT (% weight loss; 7.6% versus 4.8%, 95% CI, 1.36–12.1, *p* = 0.045) and clinically important more weight at 3-months post-RT (− 11.6% versus − 8.2%, 95% CI, − 0.6–7.5, *p* = 0.096).

### Feeding tube dependency

Feeding tube dependency was reported in three studies [36, 38, 39] (pooled *n* = 380). All studies reported varying definition of feeding tube dependency (Table 1). One of the studies [38] used gastrostomy dependency as a surrogate marker for swallowing ability, not analysed as a primary outcome. Overall, these studies suggested patients with OPC+ may experience lower feeding tube dependency rates at all reported time points (Table 1) compared to patients with OPC− (GRADE certainty of evidence low, downgraded due to serious bias and inconsistency). Bledsoe et al. 2013[36] (total *n* = 121, OPC+ *n* = 97 versus OPC − *n* = 24) found no patient with OPC+ had a gastrostomy tube still in situ at 6-months post-RT compared to 24% of patients with OPC− (*p* < 0.001), although the number of patients actively using their gastrostomy was not reported. Marzouki et al. [38] (total *n* = 112; p16+ *n* = 63 versus p16- *n* = 49 (p16+ used as a surrogate for HPV+ status) found p16+ status did not have a significant effect on requirement for gastrostomy feeding in patients undergoing major resection with free-flap construction to maintain daily caloric (energy) needs at 6- (*p* = 0.084), or 12-months post-surgery (*p* = 0.23). Naik et al. [39] (Total *n* = 147; OPC+ *n* = 130 versus OPC− *n* = 17) did not define gastrostomy dependency but reported patients with OPC+ had a lower incidence of tube dependency at 2-years post-chemoradiotherapy (1.6% versus 12.5%, *p* = 0.06) versus patients with OPC− disease.

### Feeding tube timing of placement (prophylactic or reactive) and/or utilisation

Three retrospective cohort studies [13, 19, 23] reported on feeding tube time of placement and/or utilisation during active treatment (pooled *n* = 255). One study [40] also investigated rates of prophylactic versus reactive feeding tube placement in relation to HPV status. These studies suggested patients with OPC+ may have a higher rate of reactive feeding tube placement and may require a longer duration of feeding tube use compared to patients with OPC− . GRADE certainty of evidence was low, downgraded due to serious bias and imprecision. Harrowfield et al. [19] demonstrated patients with OPC+ were more likely to require reactive nasogastric feeding in relation to poor oral intake after treatment initiation compared to patients with OPC− disease (*n* = 21 (30%) versus *n* = 3 (23%) respectively); however, this did not reach statistical significance (OR 0.75, 95%CI, 0.22–0.26, *p* = 0.65). Vangelov et al. [40] demonstrated more patients with OPC+ required a feeding tube (*n* = 43/68, 63%) compared to OPC− and HPV unknown status (*n* = 18/32, 56%). Of the 43 patients with OPC+ who received a feeding tube, 27 (63%) had the tube placed reactively and 16 (24%) prophylactically. All patients with OPC+ with a reactive feeding tube and 94% of patients with a prophylactic feeding tube experienced critical weight loss (defined as ± 5% weight loss). Patients with OPC+ with prophylactic feeding tubes had significantly higher mean percentage weight loss compared to patients with OPC− or HPV unknown status with a prophylactic feeding tube (8.6% versus 3.9%, 95% CI, 1.7–7.6, *p* = 0.003). When comparing prophylactic to reactive feeding tubes, there was no significant difference in the meantime for feeding tube utilisation for patients with OPC + (71 versus 83 days, *p* = 0.093). Vatca et al. [23] demonstrated patients with OPC+ displayed a clinically important longer duration of gastrostomy use during treatment (48.3 days) versus patients with OPC− disease (mean days 165.4 ± 163.4 versus 117.1 ± 98.2; *p* = 0.39).

### Nutritional (energy and/or protein) intake

Two retrospective cohort studies [19, 39] reported nutrition intake of patients during active treatment (pooled *n* = 230). Overall, these studies suggest no difference in nutritional intake with patients with OPC+ being more likely to resume a normal diet post-treatment compared to patients with OPC− . GRADE certainty of evidence was very low, downgraded due to serious bias and very serious imprecision. Harrowfield et al. [19] assessed dietary adequacy using the dietary intake component of the validated Patient-Generated Subjective Global Assessment (PG-SGA) tool [41], and found no significant differences in self-reported dietary adequacy based on HPV status. Naik et al. [39] reported that at 2-year post-chemoradiotherapy, patients with OPC+ experienced a lower incidence of limited diet (8.6% versus 33.3%, *p* = 0.014) versus patients with OPC− . Patients with OPC+ had more frequently resumed a normal diet (87% versus 65%) and had lower rates of limited diet (9% versus 18%) at last follow-up (median 55 months) versus patients with OPC− disease (*p* = 0.02), thereby suggested patients with OPC+ have more favourable swallowing outcomes. However, more patients with OPC− were treated with 5FU and/or twice-daily RT, which may have contributed to increased treatment toxicities in this cohort.

### Nutritional status

One retrospective cohort study (pooled *n* = 83) [19] assessed nutritional status change (i.e., malnutrition development) in patients with OPC based on HPV status. The authors utilised the validated PG-SGA tool to assess both malnutrition presence (PG-SGA category B/C change) and malnutrition severity (PG-SGA score change (mean ± SD)). This study found no significant differences between patients with OPC+ and OPC− for PG-SGA category (i.e., malnutrition presence; *p* = 0.30) or score change (i.e., malnutrition severity; *p* = 0.44) at any time point. GRADE certainty of evidence was very low, downgraded due to serious bias and very serious imprecision. At 3-months post-treatment, there was no difference for patients with OPC+ assessed as having PG-SGA category B/C (moderate-severe) malnutrition (*n* = 30; 43%) versus OPC− (*n* = 5; 38%; *p* = 0.30). Although two other studies [23, 40] were identified stating they reported on nutritional status change, both studies used weight change as a surrogate for nutritional status and were therefore not included.

## Discussion

Despite the rising epidemic of patients diagnosed with OPC+ , this is the first systematic review to our knowledge that has examined nutrition outcomes specific to patients with OPC+ undergoing any treatment modality when compared solely to patients with OPC− . Key findings demonstrate patients with OPC+ status may experience greater weight loss during and post treatment, have higher utilisation of reactive feeding tubes, and may have lower feeding tube dependency rates compared to patients with OPC− . It is uncertain whether nutritional intake and nutritional status differed between populations. This review highlights the limited research currently available investigating nutrition outcomes for the changing landscape of OPC, and suggests patients with OPC+ may have greater acute (i.e., more weight loss and requirement for reactive feeding tube placement) but lower chronic nutrition needs (i.e., lower feeding tube dependency) than those with OPC− disease.

The higher rates of critical weight loss reported for patients with OPC+ versus OPC− disease is concerning, considering the markedly improved prognosis and potential chronic impacts on QoL [42]. The causes are likely multifactorial, expected to include the same challenges as previously well established for the OPC− population [43, 44]. However, given the distinct clinical and pathological differences, additional unique barriers specific to this population may be present [3, 19, 40]. Acute toxicities experienced by patients with OPC+  during treatment may have contributed to the increased rates of weight loss reported [28]. Vatca et al. [23] supports this theory, attributing the higher rates of weight loss seen for patients with OPC + to increased patient-reported burden from mucositis, despite the significantly higher tumour staging for OPC− patients at baseline. The impact of a higher perceived intensity of radiation-induced pain [45], potentially due to the lower smoking rates seen for OPC+ versus OPC− [46], and higher levels of fatigue [47] reported for the OPC+ population may also increase this risk of critical weight loss further. Given patients with OPC+ often report higher QoL and minimal symptoms at diagnosis, they may perceive a larger decrease in their QoL when the impacts of acute toxicities become apparent, increasing patients distress, compared to those with OPC− disease already experiencing tumour burden at diagnosis [18, 19, 48]. It has been demonstrated that as the acuity of treatment side effects worsen, impacting on nutritional intake, weight loss for many becomes uncontrolled, worsening patient-reported distress [49]. Although no psychological interventions specific to the OPC+ population are known to have been conducted, a recent study which included distress screening and referral [50, 51] with a high proportion of patients with OPC (mixed OPC+ and OPC− ; 56%) demonstrated improved adherence to nutritional recommendations, nutritional outcomes, and QoL. Further research considering multi-disciplinary (MDT) interventions is required given these promising results [50, 51].

Tube (enteral) feeding is a commonly utilised nutrition intervention in patients with HNC to attenuate nutritional decline, regardless of HPV status [7, 8, 40]. A recent review of five studies (*n* = 298) that included heterogenous patients with HNC undergoing radiotherapy ± chemotherapy demonstrated patients who received a prophylactic tube/feeding were less likely to experience short-term weight loss and improved short-term QoL versus those with reactive placements/feeding [52]. Regardless, the optimal timing of insertion (prophylactic versus reactive) and time to commencement of tube feeds in clinical practice for the heterogenous HNC population remains highly variable and controversial [19, 44]. Studies of patients with OPC+ in this review had a higher rate of reactive tube placement [19, 23, 40] compared to patients with OPC− [23]. This suggests a higher proportion of patients with OPC + are unable to maintain adequate nutritional requirements orally during treatment. The reasons for this are likely multifactorial, heightened by the different demographic and clinical presentations of OPC+ populations. This may decrease patient and clinician concern regarding weight loss and reduce nutritional guideline adherence, in particular prophylactic insertion recommendations [3, 19]. Patient-reported barriers to nutrition care and tube feeding have been reported in the literature and can include uncontrolled nutrition impact symptoms, psychosocial and economic barriers, and environmental factors [19, 43, 49, 50, 53]. A lack of patient knowledge regarding the importance of optimising nutritional status may also be present, as it is known many report weight loss to be a beneficial side-effect of treatment [53, 54]. This knowledge gap may be being exacerbated by MDT perceptions, given as a recent study demonstrated patients with HNC consistently reported receiving conflicting information from the MDT regarding weight loss and tube feeding, contradicting best evidence [54]. This could contribute to confusion and ultimately impede informed decision-making by patients, exacerbating nutritional decline [54]. Ensuring that the educational needs of patients with OPC+ is addressed, particularly prior to treatment commencement, may improve adherence and subsequent nutrition outcomes for patients with OPC+ overall [43, 53, 54].

Clinician awareness and knowledge regarding nutrition and tube feeding for patients with OPC+ should therefore be recognised as a key part for improving patient outcomes. Clinicians may be reluctant to insert prophylactic feeding tubes in OPC+ patients who are younger, more overweight and/or obese, and display higher motivation to continue with oral intake for as long as possible compared to OPC− populations [3, 19, 40]. Harrowfield et al. [19] demonstrated that although 87% of patients with OPC+ had ± 5% weight loss during treatment, only 64% had a feeding tube inserted. Similarly, Vangelov et al. [40] showed 94% of patients with OPC+ had > 5% weight loss, although only 64% had a feeding tube inserted. Despite 12 patients with OPC+ presenting with ≥ 5% weight loss at diagnosis, only four had a prophylactic tube inserted as per their institutional practice [40]. The studies suggest that the number of patients with OPC+ who likely required and would have benefited from earlier tube feeding was high, and a prophylactic approach may still be a relevant and appropriate mode of nutrition intervention. This is consistent with Brown et al. who found heterogenous patients with HPV-positive HNC (oral and oropharyngeal) had 4.4 times greater odds of requiring a proactive gastrostomy than those with HPV-negative disease [8]. The evolution of primary transoral robotic surgery (TORS) for select low-risk patients, inclusive of OPC+ , will likely play a role in influencing feeding tube requirement and use for this population [55]. Recent studies using this treatment modality in heterogenous HNC populations have demonstrated improved weight maintenance with minimal tube feeding requirement rates [55–57]. However, a recent meta-analysis conducted solely in patients with OPC+ failed to show statistically significant difference between surgery (inclusive of TORS) with adjuvant therapy compared to chemoradiotherapy with cisplatin at 12 (*p* = 0.37) or 24 to 36 months (*p* = 0.06) [58]. Relatedly, Dziegielewski et al. [37] found OPC+ status was not a prognosticative factor for feeding tube dependency post-TORS (OPC+ OR 0.8 (95% CI, 0.2–2.6%, *p* = 0.68)). The reduced long-term feeding tube dependency rates seen for patients with OPC+ despite prophylactic placement also requires further investigation, as it could be hypothesised that an improved recovery capacity post-RT is seen for patients with OPC+ compared to those with OPC− disease [48], since less concomitant risk factors (such as smoking and/or alcohol use) are often seen which may increase clinician confidence for supporting tube feeding interventions [14]. Future research should therefore consider predictive factors, treatment modality, and optimal timing of tube placement for the OPC+ population, particularly as rates of long-term feeding tube dependency were low.

Patients with OPC+ may be more likely to be well-nourished and in the overweight/obese BMI categories at diagnosis compared to patients who are OPC− ; however, this does not appear to negate nutritional decline [13, 19, 40]. Sarcopenia, defined as a loss of muscle function and strength [59], is a current key focus of oncological research, as sarcopenia development during treatment is recognised as an independent predictor of reduced survival [60–62]. Pre-treatment sarcopenia prevalence in patients with OPC+ has been shown to range from 20 to 55.6% despite a higher presenting BMI [63–66]. The presence of concurrent sarcopenia in patients who are overweight/obese is an often overlooked, condition, despite higher rates of mortality and treatment-related complications [60, 67, 68]. As body surface area calculations used to scale chemotherapy dosing do not discern for variations in body composition[69], the potential increased exposure to chemotherapeutic dosages may be a contributing factor for increased toxicities for those with sarcopenic obesity [60, 67]. The higher BMI at diagnosis often seen for patients with OPC+ may therefore be masking an underlying sarcopenia [3, 18, 19]. Future research is warranted to both assess and fully elucidate the prognostic significance sarcopenic obesity has for the OPC+ population.

Strengths of this review include the strict eligibility criteria of only including peer-reviewed studies that compared solely patients with OPC+ to patients with OPC− , the rigorous application of bias assessment, and use of GRADE. The clinical diversity present between the identified studies (Table 1) with regard to variability in study populations (i.e., stage III–IVb OPC versus all TNM stages; use of p16 as a surrogate marker for HPV status), treatment regimens and/or agents used (i.e., RT (3D-conformal versus Intensity-Modulated Radiation Therapy) ± CT (fluorouracil with cisplatin or high-dose cisplatin ± cetuximab) ± surgical intervention), and different nutrition outcomes reported and their measurement (i.e., definition and measurement of feeding tube dependency) was high. These factors coupled with the retrospective nature of the studies identified, decreased overall certainty, and limited the ability to perform a more robust meta-analysis. Additionally, the long recruitment periods reported by some of the studies may have led to mixed AJCC classification systems being used for diagnosis and staging [16].

## Conclusion

This review demonstrates that weight loss and requirement for reactive tube feeding is high and gastrostomy dependency low for the rising prevalence of younger patients diagnosed with OPC+ . Despite the significantly improved prognosis, as there are no nutrition guidelines specific to this unique subset of patients to help guide clinical care, the risks of suboptimal health and patient-centred outcomes and negative impacts on long-term QoL remains high. Further high-quality research is needed to understand nutritional care practices for patients with OPC+ , to allow this population to achieve optimal positive health outcomes carried into survivorship.

## Supplementary Information

Below is the link to the electronic supplementary material.

Supplementary file1 (DOCX 13 KB)

Supplementary file2 (DOCX 13 KB)

## Data Availability

As this is a systematic literature review, the authors declare no control of the primary data presented.
