# Supplementary material for: The changing face of head and neck cancer: are patients with human papillomavirus-positive disease at greater nutritional risk? A systematic review
Source: Support Care Cancer. 2022 Apr 27;30(9):7191–204. doi: 10.1007/s00520-022-07056-9 (PMC9385807; doi:10.1007/s00520-022-07056-9)
Supplement: Supplementary file 1 — Supplementary file1 (DOCX 13 KB) [file 520_2022_7056_MOESM1_ESM.docx]

Supplementary Table 1. PICO Statement

| P –patients >18 years with human papillomavirus-positive oropharyngeal head and neck cancer |
| --- |
| I – conventional cancer treatment (any modality) of curative intent |
| C –patients >18 years with human papillomavirus-negative oropharyngeal head and neck cancer undergoing conventional cancer treatment (any modality) of curative intent |
| O – weight change, malnutrition presence and/or incidence, feeding tube time of placement and/or utilisation, feeding tube dependency, and nutritional (energy and/or protein) intake. |
