# Supplementary material for: The changing face of head and neck cancer: are patients with human papillomavirus-positive disease at greater nutritional risk? A systematic review
Source: Support Care Cancer. 2022 Apr 27;30(9):7191–204. doi: 10.1007/s00520-022-07056-9 (PMC9385807; doi:10.1007/s00520-022-07056-9)
Supplement: Supplementary file 2 — Supplementary file2 (DOCX 13 KB) [file 520_2022_7056_MOESM2_ESM.docx]

Supplementary Table 2. PubMed Search Strategy

| ((("papillomaviridae"[MeSH Terms] OR "Papillomavirus Infections"[MeSH Terms:noexp]) OR ("human papillomavirus"[Text Word] OR "HPV"[Text Word] OR "HPV16"[Text Word] OR "p16"[Text Word] OR "HPV18"[Text Word] OR "HPV 16"[Text Word] OR "HPV 18"[Text Word] OR "papillomaviridae"[Text Word])) AND (("Head and Neck Neoplasms"[MeSH Terms]) OR (("cancer*"[Text Word] OR "neoplasm*"[Text Word] OR "carcinoma*"[Text Word] OR "tumour*"[Text Word] OR "tumor*"[Text Word] OR "malignanc*"[Text Word]) AND ("head"[Text Word] OR "neck"[Text Word] OR "oropharyn*"[Text Word] OR "nasopharyn*"[Text Word] OR "hypopharyn*"[Text Word] OR "oral"[Text Word]))))))) AND (("body weight changes"[MeSH Terms] OR "Health Status"[MeSH Terms] OR "nutritional support"[MeSH Terms] OR "nutrition therapy"[MeSH Terms] OR "quality of life"[MeSH Terms] OR "sarcopenia"[MeSH Terms] OR “cachexia”[MeSH Terms]) OR ("malnutrition"[Text Word] OR "weight*"[Text Word] OR "feed*"[Text Word] OR "nutrition*"[Text Word] OR "diet*"[Text Word] OR "enteral*"[Text Word] OR "parenteral*"[Text Word] OR "tube"[Text Word] OR "PEG"[Text Word] OR “reactive”[Text Word] OR “prophylactic”[Text Word] OR "gastrostom*"[Text Word] OR "nasogastric*"[Text Word] OR "tube"[Text Word] OR "interruptions"[Text Word] OR "mal*"[Text Word] |
| --- |
